# Supplementary material for: Pathogenomic analysis and characterization of Pasteurella multocida strains recovered from human infections
Source: Microbiol Spectr. 2024 Mar 1;12(4):e03805-23. doi: 10.1128/spectrum.03805-23 (PMC10986470; doi:10.1128/spectrum.03805-23)
Supplement: Supplemental Material 1 — Additional materials and methods, Tables S1 to S6, and Figures S1 to S5. [file spectrum.03805-23-s0001.docx]

## Supplemental material 1 for Pathogenomic analysis and characterisation of *Pasteurella multocida* strains recovered from human infections

Additional methods

*In silico* confirmation of species and identification of subspecies

Isolates were confirmed as *P. multocida* species using average nucleotide identity (ANI), compared to *P. multocida* type strain NCTC 10322, determined using fastANI. ANI has previously been used for species identification, with an ANI value >96 used to call two strains the same species (1). All isolates had an ANI value >96 compared to strain NCTC 10322 (Table S3). ANI can also differentiate subspecies, with an ANI value >98 used to call two strains the same subspecies (2, 3). As such, to identify the subspecies of *P. multocida* strains, we used ANI value comparisons against strains representing each subspecies, namely NCTC 10322 (subsp. *multocida*), NCTC 10204 (subsp. *gallicida*) and NCTC 11995 (subsp. *septica*) for identification of subsp. *septica* strains. ANI comparisons allowed for the identification of subsp. *septica* strains, but ANI value between strain NCTC 10322 (subsp. *multocida*) and NCTC 10204 (subsp. *gallicida*) was >98, so strains belonging to these subspecies could not be differentiated using this method. Instead, to identify subsp. *gallicida* strains we searched genomes for the presence of *gatD*, a gene encoding a galactitol dehydrogenase required for galactitol utilisation, that is unique to subsp. *gallicida* strains (4). Of the 402 genomes included in this study, only four draft genomes and two complete genomes were typed as subsp. *gallicida*. Given strains NCTC 10322 and NCTC 10204 had an ANI value >98, and strains typed as subsp. *gallicida* were present throughout the core-genome phylogeny (Fig. 1), it is likely that subsp. *multocida* and *gallicida* are the same subspecies.

The *P. multocida* virulence factor and antibiotic resistance database

Initially, the *P. multocida* strains used in this study were searched against the curated databases ARG-ANNOT and virulence factor database using ABRicate; however, these searches yielded few results with several gaps, making functional predictions of matches difficult. To overcome this, we compiled a curated database of previously identified virulence factor and antibiotic resistance genes from *P. multocida* (named the PastyVRDB). Genes were included in the PastyVRDB if there was direct evidence of function, or identity to known virulence factor or antibiotic resistance genes. The full PastyVRDB is provided as Supplemental material 4, with metadata in Table S6. Several capsule and LPS biosynthesis genes present in different loci have high nucleotide and amino acid identity. As such, searches against the PastyVRDB resulted in some strains having matches to genes from multiple capsule or LPS types; however, this was only for genes with high identity.

Roary pan-genome analysis and Scoary analysis for identification of over- or under-represented genes

A pan-genome analysis was performed using Roary to generate a core-genome alignment that was then used to generate the *P. multocida* phylogeny. The Roary analysis for the 402 *P. multocida* strains used in this study gave a core-genome of 1,681 genes and a pan-genome of 7,864 genes. Scoary analysis was specifically used to identify genes over- or under-represented in *P. multocida* strains isolated from humans, or from *P. multocida* subsp. *septica* and capsule type A strains. To avoid incomplete genomes impacting Scoary analysis, only complete/closed genomes were used for this analysis. Additionally, the sequence of any genes present or absent in the *P. multocida* subsp. *septica* strains were then searched for matches against the pan-genome reference list generated by Roary. Genes identified by Scoary that had a >90% nucleotide identity to the reference strain from another group were taken to be homologs; this avoided gene groups likely encoding proteins with the same function as being incorrectly identified as over- or under-represented for a particular trait.

Supplemental tables and figures

**TABLE S1:** Bacterial strains used in this study

| Strain | Description | Reference |
| --- | --- | --- |
| VP161 | Avian isolate strain, serotype A:L1 | (5) |
| AL3574 | VP161 *hyaD* TargeTron mutant, intron inserted between *hyaD* nucleotides 1483-1484; Kan^R^, acapsular | (6) |
| Past1 | Human blood culture isolate, recovered in Victoria, Australia | This study |
| Past3 | Human blood culture isolate, recovered in Victoria, Australia | This study |
| Past4 | Human blood culture isolate, recovered in Victoria, Australia | This study |
| Past5 | Human blood culture isolate, recovered in Victoria, Australia | This study |
| Past6 | Human peritoneal dialysis isolate, recovered in Victoria, Australia | This study |
| Past7 | Human peritoneal dialysis isolate, recovered in Victoria, Australia | This study |
| Past9 | Human peritoneal dialysis isolate, recovered in Victoria, Australia | This study |
| Past10 | Human cat bite isolate, recovered in Victoria, Australia | This study |
| Past11 | Human skin tissue isolate, recovered in Victoria, Australia | This study |
| Past13 | Human blood culture isolate, recovered in Victoria, Australia | This study |
| Past15 | Human respiratory tract isolate, recovered in Victoria, Australia | This study |
| Past18 | Human cat bite isolate, recovered in Victoria, Australia | This study |
| Past19 | Human cat bite abscess isolate, recovered in Victoria, Australia | This study |
| Past22 | Human cat bite isolate, recovered in Victoria, Australia | This study |
| Past23 | Human peritoneal dialysis isolate, recovered in Victoria, Australia | This study |
| Past26 | Human skin tissue isolate, recovered in Victoria, Australia | This study |
| Past28 | Human cat bite isolate, recovered in Victoria, Australia | This study |
| Past29 | Human blood culture isolate, recovered in Victoria, Australia | This study |
| Past30 | Human cat bite isolate, recovered in Victoria, Australia | This study |
| Past31 | Human skin tissue isolate, recovered in Victoria, Australia | This study |
| Past33 | Human skin tissue isolate, recovered in Victoria, Australia | This study |
| Past34 | Human dog bite isolate, recovered in Victoria, Australia | This study |
| Pm1476 | Cat upper respiratory tract isolate, recovered in Queensland, Australia | This study |
| Pm1612 | Cat upper respiratory tract isolate, recovered in Queensland, Australia | This study |
| Pm1613 | Cat upper respiratory tract isolate, recovered in Queensland, Australia | This study |
| Pm1616 | Cat upper respiratory tract isolate, recovered in Queensland, Australia | This study |
| Pm1617 | Cat upper respiratory tract isolate, recovered in Queensland, Australia | This study |
| Pm1618 | Cat upper respiratory tract isolate, recovered in Queensland, Australia | This study |
| Pm1620 | Cat upper respiratory tract isolate, recovered in Queensland, Australia | This study |
| Pm1621 | Cat upper respiratory tract isolate, recovered in Queensland, Australia | This study |
| Pm1622 | Cat upper respiratory tract isolate, recovered in Queensland, Australia | This study |
| Pmc-A | Cat upper respiratory tract isolate, recovered in Queensland, Australia | This study |
| Pmc-B | Cat upper respiratory tract isolate, recovered in Queensland, Australia | This study |
| Pmc-C | Cat upper respiratory tract isolate, recovered in Queensland, Australia | This study |
| PI31 | Dog wound isolate, recovered in Queensland, Australia | This study |
| PI32 | Dog tissue isolate, recovered in Queensland, Australia | This study |

**TABLE S2:** *P. multocida* isolate sequencing and genome assembly quality control data

| Strain | Genome size | Sequencing depth | Contigs^1^ | N50 | L50 | GC content (%) | Number of ORFs |
| --- | --- | --- | --- | --- | --- | --- | --- |
| Past1 | 2,290,030 | 81x | 30 | 150,782 | 6 | 40.40 | 2159 |
| Past3 | 2,308,299 | 75x | 2^C^ | 2,305,893 | 1 | 40.42 | 2187 |
| Past4 | 2,222,762 | 72x | 43 | 91,867 | 9 | 40.52 | 2079 |
| Past5 | 2,209,709 | 80x | 22 | 133,647 | 5 | 40.42 | 2088 |
| Past6 | 2,222,806 | 73x | 3^C^ | 2,217,586 | 1 | 40.58 | 2092 |
| Past7 | 2,312,025 | 75x | 22 | 151,557 | 4 | 40.38 | 2217 |
| Past9 | 2,419,174 | 172x | 1^C^ | 2,419,174 | 1 | 40.55 | 2343 |
| Past10 | 2,442,576 | 69x | 48 | 90,591 | 9 | 40.25 | 2374 |
| Past11 | 2,287,048 | 71x | 41 | 101,177 | 8 | 40.44 | 2174 |
| Past13 | 2,284,711 | 73x | 27 | 155,275 | 5 | 40.37 | 2173 |
| Past15 | 2,473,596 | 126x | 4 | 2,279,351 | 1 | 40.30 | 2414 |
| Past18 | 2,272,683 | 70x | 28 | 161,774 | 6 | 40.36 | 2157 |
| Past19 | 2,162,151 | 67x | 32 | 113,211 | 7 | 40.40 | 2047 |
| Past22 | 2,264,618 | 29x | 63 | 101,116 | 7 | 40.48 | 2149 |
| Past23 | 2,308,982 | 27x | 62 | 70,506 | 12 | 40.43 | 2186 |
| Past26 | 2,180,995 | 43x | 54 | 82,630 | 10 | 40.61 | 2053 |
| Past28 | 2,248,839 | 96x | 34 | 139,426 | 7 | 40.51 | 2131 |
| Past29 | 2,274,526 | 151x | 2^C^ | 2,272,271 | 1 | 40.43 | 2135 |
| Past30 | 2,227,555 | 34x | 24 | 173,135 | 5 | 40.42 | 2079 |
| Past31 | 2,254,775 | 38x | 49 | 92,486 | 9 | 40.58 | 2141 |
| Past33 | 2,312,722 | 58x | 2^C^ | 2,309,199 | 1 | 40.42 | 2196 |
| Past34 | 2,360,301 | 43x | 53 | 85,958 | 8 | 40.58 | 2264 |
| Pm1476 | 2,306,050 | 29x | 59 | 161,858 | 6 | 40.5 | 2196 |
| Pm1612 | 2,189,281 | 45x | 31 | 108,374 | 6 | 40.38 | 2040 |
| Pm1613 | 2,193,562 | 31x | 23 | 192,146 | 4 | 40.35 | 2047 |
| Pm1616 | 2,216,246 | 43x | 41 | 121,108 | 7 | 40.34 | 2084 |
| Pm1617 | 2,240,225 | 23x | 64 | 69,675 | 11 | 40.44 | 2119 |
| Pm1618 | 2,261,254 | 92x | 1^C^ | 2,261,254 | 1 | 40.52 | 2145 |
| Pm1620 | 2,196,796 | 48x | 21 | 167,963 | 4 | 40.34 | 2047 |
| Pm1621 | 2,279,711 | 98x | 1^C^ | 2,279,711 | 1 | 40.37 | 2141 |
| Pm1622 | 2,227,463 | 29x | 28 | 196,480 | 4 | 40.44 | 2111 |
| Pmc-A | 2,279,063 | 36x | 25 | 318,478 | 3 | 40.43 | 2183 |
| Pmc-B | 2,268,061 | 54x | 29 | 185,538 | 5 | 40.47 | 2167 |
| Pmc-C | 2,277,767 | 28x | 25 | 311,494 | 3 | 40.43 | 2182 |
| PI31 | 2,228,956 | 53x | 25 | 219,012 | 3 | 40.42 | 2118 |
| PI32 | 2,240,863 | 49x | 25 | 207,556 | 4 | 40.43 | 2110 |

^1^Only contigs >500 nucleotides in length were included in the count

^C^Closed genomes generated by Nanopore sequencing, all other genomes list are draft/incomplete.

**TABLE S3.** Average nucleotide identity (ANI) score for *P. multocida* isolates sequenced in this study compared to *P. multocida* subspecies reference strains NCTC 11995 (subsp. *septica*), NCTC 10322 (subsp. *multocida*), and NCTC 1204 (subsp. *gallicida*)*.* ANI values were generated using fastANI.

| Strain | ANI value to subsp. *multocida* strain NCTC 10322 | ANI value to subsp. *gallicida* strain NCTC 10204 | ANI value to subsp. *septica* strain NCTC 11995 |
| --- | --- | --- | --- |
| Past1 | 96.79 | 96.74 | 99.00 |
| Past3 | 98.60 | 98.59 | 97.11 |
| Past4 | 96.95 | 96.96 | 98.96 |
| Past5 | 96.89 | 96.93 | 98.97 |
| Past6 | 96.83 | 96.87 | 99.06 |
| Past7 | 96.84 | 96.71 | 98.98 |
| Past9 | 96.99 | 96.81 | 98.84 |
| Past10 | 96.83 | 96.81 | 98.84 |
| Past11 | 96.94 | 96.95 | 99.05 |
| Past13 | 96.85 | 96.73 | 98.91 |
| Past15 | 98.81 | 98.81 | 96.94 |
| Past18 | 96.93 | 96.92 | 98.88 |
| Past19 | 97.00 | 96.96 | 98.85 |
| Past22 | 97.05 | 97.00 | 98.87 |
| Past23 | 96.75 | 96.88 | 98.97 |
| Past26 | 96.98 | 96.96 | 98.95 |
| Past28 | 96.89 | 96.89 | 98.99 |
| Past29 | 98.33 | 98.25 | 97.53 |
| Past30 | 96.88 | 96.98 | 98.93 |
| Past31 | 96.89 | 96.84 | 98.90 |
| Past33 | 98.70 | 98.72 | 96.90 |
| Past34 | 96.88 | 96.80 | 98.96 |
| Pm1476 | 96.85 | 96.91 | 99.01 |
| Pm1612 | 98.89 | 98.92 | 96.92 |
| Pm1613 | 98.84 | 98.85 | 96.88 |
| Pm1616 | 98.82 | 98.85 | 96.95 |
| Pm1617 | 98.71 | 98.83 | 96.95 |
| Pm1618 | 96.84 | 96.86 | 98.94 |
| Pm1620 | 98.87 | 98.82 | 96.89 |
| Pm1621 | 98.77 | 98.87 | 96.96 |
| Pm1622 | 96.87 | 96.87 | 98.96 |
| Pmc-A | 96.82 | 96.87 | 98.95 |
| Pmc-B | 96.78 | 96.92 | 98.97 |
| Pmc-C | 96.72 | 96.85 | 98.97 |
| PI31 | 96.87 | 96.93 | 98.97 |
| PI32 | 98.75 | 98.81 | 96.87 |

**TABLE S4.** Plasmids identified in the human, cat, and dog *P. multocida* isolates sequenced in this study using hybrid Nanopore/Illumina assemblies and PlasClass.

| Plasmid | Genbank accession number^1^ | Strains harbouring plasmid | Size (bp)^2^ | Best PLSDB match | Best NCBI match^3^ | Predicted copy number |
| --- | --- | --- | --- | --- | --- | --- |
| pBAC1 | JAVSKG010000028, JAVSKE010000021, CP133640, JAVSKD010000021, JAVSKA010000026 | Past1  Past5  Past6  Past7  Past13 | 3,682 | NZ_CP020404 | CP097619, 99% nt identity with 96% coverage | 24 |
| pAL1941 | CP133643 | Past3 | 2,406 | NA | NA | 41 |
| pAL1942 | CP133641, CP133637 | Past6  Past29 | 2,255 | NZ_U51470 | CP085872, 99.8% nt identity with 100% coverage | 10 |
| pAL1943 | CP133635 | Past33 | 3,523 | NA | JQ319773, 100% nt identity with 84% coverage | 8 |
| pAL1944 | JAVSKC010000035, JAVSKC010000038, JAVSKC010000041 | Past10 | NA | NA | CP049757^4^ all contigs had at least >93% identity with >95% coverage | NA |

^1^The assembly for pAL1944 is split into three contigs

^2^NA - not applicable

^3^nt – nucleotide

^4^All three pAL1944 contigs matched to accession number CP049757

**TABLE S5.** Prophages in human, cat, and dog *P. multocida* isolates sequenced in this study identified using PhiSpy.

| Phage | Accession number | Strains containing prophage | Size (bp) | Number of open reading frames |
| --- | --- | --- | --- | --- |
| Pmp1 | OR544132 | Past1 | 14,066 | 26 |
| Pmp2 | OR544133 | Past6 | 25,897 | 37 |
| Pmp3 | OR544134 | Past23 | 39,677 | 29 |
| Pmp4 | OR544135 | Past30 | 20,473 | 27 |
| Pmp5 | OR544136 | Past3 | 22,544 | 32 |
| Pmp6 | OR544137 | Past34 | 30,190 | 40 |
| Pmp7 | OR544138 | Past29 | 10,714 | 17 |
| Pmp8 | OR544139 | Pm1621 | 15,321 | 23 |
| Pmp9 | OR544140 | Past9 | 20,771 | 30 |
| Pmp10 | OR544127 | Pas18 | 18,415 | 35 |
| Pmp11 | OR544128 | Past3 | 32,839 | 43 |
| Pmp12 | OR544129 | Past22 | 20,032 | 30 |
| Pmp13 | OR544130 | Past33 | 38,501 | 68 |
| Pmp14 | OR544131 | Pm1618, Pm1622,  Pmc-A, Pmc-B, PI31 | 43,801 | 58 |

**Table S6.** *Pasteurella multocida* virulence factor and resistance database metadata.

| Gene | Strain or plasmid | Genbank accession | Function | Reference |
| --- | --- | --- | --- | --- |
| *phyA* | X73 | AF067175 | Capsule attachment | (7) |
| *phyB* | X73 | AF067175 | Capsule attachment | (7) |
| *hyaE* | X73 | AF067175 | Type A capsule biosynthesis | (7) |
| *hyaD* | X73 | AF067175 | Type A capsule biosynthesis | (7) |
| *hyaC* | X73 | AF067175 | Type A capsule biosynthesis | (7) |
| *hyaB* | X73 | AF067175 | Type A capsule biosynthesis | (7) |
| *dcbE* | HN06 | CP003313.1 | Type D capsule biosynthesis | (8) |
| *dcbF* | HN06 | CP003313.1 | Type D capsule biosynthesis | (8) |
| *dcbC* | HN06 | CP003313.1 | Type D capsule biosynthesis | (8) |
| *dcbB* | HN06 | CP003313.1 | Type D capsule biosynthesis | (8) |
| *fcbE* | P4218 | AF302467 | Type F capsule biosynthesis | (7) |
| *fcbD* | P4218 | AF302467 | Type F capsule biosynthesis | (7) |
| *fcbC* | P4218 | AF302467 | Type F capsule biosynthesis | (7) |
| *fcbB* | P4218 | AF302467 | Type F capsule biosynthesis | (7) |
| *hexD* | X73 | AF067175 | Capsule ABC-transporter | (7) |
| *hexC* | X73 | AF067175 | Capsule ABC-transporter | (7) |
| *hexB* | X73 | AF067175 | Capsule ABC-transporter | (7) |
| *hexA* | X73 | AF067175 | Capsule ABC-transporter | (7) |
| *cexD* | M1404 | AF169324 | Capsule export | (9) |
| *cexC* | M1404 | AF169324 | Capsule export | (9) |
| *cexB* | M1404 | AF169324 | Capsule export | (9) |
| *cexA* | M1404 | AF169324 | Capsule export | (9) |
| *lipA* | M1404 | AF169324 | Capsule attachment | (9) |
| *lipB* | M1404 | AF169324 | Capsule attachment | (9) |
| *bcbA* | M1404 | AF169324 | Type B capsule biosynthesis | (9) |
| *bcbB* | M1404 | AF169324 | Type B capsule biosynthesis | (9) |
| *bcbC* | M1404 | AF169324 | Type B capsule biosynthesis | (9) |
| *bcbD* | M1404 | AF169324 | Type B capsule biosynthesis | (9) |
| *bcbE* | M1404 | AF169324 | Type B capsule biosynthesis | (9) |
| *bcbF* | M1404 | AF169324 | Type B capsule biosynthesis | (9) |
| *bcbG* | M1404 | AF169324 | Type B capsule biosynthesis | (9) |
| *bcbH* | M1404 | AF169324 | Type B capsule biosynthesis | (9) |
| *bcbI* | M1404 | AF169324 | Type B capsule biosynthesis | (9) |
| *ecbA* | P1234 | AF302466 | Type E capsule biosynthesis | (7) |
| *ecbB* | P1234 | AF302466 | Type E capsule biosynthesis | (7) |
| *ecbJ* | P1234 | AF302466 | Type E capsule biosynthesis | (7) |
| *ebcK* | P1234 | AF302466 | Type E capsule biosynthesis | (7) |
| *ebcD* | P1234 | AF302466 | Type E capsule biosynthesis | (7) |
| *ecbE* | P1234 | AF302466 | Type E capsule biosynthesis | (7) |
| *ecbF* | P1234 | AF302466 | Type E capsule biosynthesis | (7) |
| *ecbG* | P1234 | AF302466 | Type E capsule biosynthesis | (7) |
| *ecbI* | P1234 | AF302466 | Type E capsule biosynthesis | (7) |
| *grxD* | Pm70 | AE004439 | Flanking capsule biosynthesis region^1^ | (10) |
| DUF441 | Pm70 | AE004439 | Flanking capsule biosynthesis region^1^ | (10) |
| *priA* | Pm70 | AE004439 | Flanking outer core LPS biosynthesis region^1^ | (10) |
| *fpg* | Pm70 | AE004439 | Flanking outer core LPS biosynthesis region^1^ | (10) |
| *kdtA* | Pm70 | AE004439 | Addition of Kdo I to the inner core, and Kdo II to the inner core of glycoform 2 | (10) |
| *hptA* | Pm70 | AE004439 | Addition of Hep I to inner core in glycoform A | (10) |
| *hptB* | Pm70 | AE004439 | Addition of Hep I to inner core in glycoform B | (10) |
| *gctB* | Pm70 | AE004439 | Addition of Glc I to the inner core | (10) |
| *kdkA* | Pm70 | AE004439 | Phosphorylation of Kdo I in inner core | (10) |
| *petK* | Pm70 | AE004439 | PEtn addition to inner core for glycoform A | (10) |
| *hptC* | Pm70 | AE004439 | Addition of Hep II to to the inner core | (10) |
| *hptD* | Pm70 | AE004439 | Addition of Hep III to the inner core | (10) |
| *lpt-3* | Pm70 | AE004439 | PEtn addition to inner core | (10) |
| *gctA* | Pm70 | AE004439 | Addition of Glc II to inner core of glycoform A | (10) |
| *pcgD* | X73 | HQ873311 | L1 outer core LPS biosynthesis | (11) |
| *pcgA* | X73 | HQ873311 | L1 outer core LPS biosynthesis | (11) |
| *pcgB* | X73 | HQ873311 | L1 outer core LPS biosynthesis | (11) |
| *pcgC* | X73 | HQ873311 | L1 outer core LPS biosynthesis | (11) |
| *gatA* | X73 | HQ873311 | L1 outer core LPS biosynthesis | (11) |
| *hptE_L1* | X73 | HQ873311 | L1 outer core LPS biosynthesis | (11) |
| *gatD* | M1404 | GQ444331 | L2 outer core LPS biosynthesis | (12) |
| *nctA* | M1404 | GQ444331 | L2 outer core LPS biosynthesis | (12) |
| *hptF* | M1404 | GQ444331 | L2 outer core LPS biosynthesis | (12) |
| *hptE_L2* | M1404 | GQ444331 | L2 outer core LPS biosynthesis | (12) |
| *natC* | P1059 | KF314825 | L3 outer core LPS biosynthesis | (13) |
| *gatG* | P1059 | KF314825 | L3 outer core LPS biosynthesis | (13) |
| *natb* | P1059 | KF314825 | L3 outer core LPS biosynthesis | (13) |
| *gatF* | P1059 | KF314825 | L3 outer core LPS biosynthesis | (13) |
| *gctC* | P1059 | KF314825 | L3 outer core LPS biosynthesis | (13) |
| *hptE_L3* | P1059 | KF314825 | L3 outer core LPS biosynthesis | (13) |
| *gatL* | P2192 | KM670447 | L4 outer core LPS biosynthesis | (14) |
| *latB* | P2192 | KM670447 | L4 outer core LPS biosynthesis | (14) |
| *gatK_L4* | P2192 | KM670447 | L4 outer core LPS biosynthesis | (14) |
| *natD* | P2192 | KM670447 | L4 outer core LPS biosynthesis | (14) |
| *natE* | P2192 | KM670447 | L4 outer core LPS biosynthesis | (14) |
| *hptG* | P2095 | JN571483.1 | L5 outer core LPS biosynthesis | (15) |
| *plbA* | P2095 | JN571483.1 | L5 outer core LPS biosynthesis | (15) |
| *rmlB* | P2095 | JN571483.1 | L5 outer core LPS biosynthesis | (15) |
| *plbB* | P2095 | JN571483.1 | L5 outer core LPS biosynthesis | (15) |
| *rhtA* | P2095 | JN571483.1 | L5 outer core LPS biosynthesis | (15) |
| *rhtB* | P2095 | JN571483.1 | L5 outer core LPS biosynthesis | (15) |
| *qdtB* | P2095 | JN571483.1 | L5 outer core LPS biosynthesis | (15) |
| *qdtD* | P2095 | JN571483.1 | L5 outer core LPS biosynthesis | (15) |
| *latA* | P2095 | JN571483.1 | L5 outer core LPS biosynthesis | (15) |
| *rmlA* | P2095 | JN571483.1 | L5 outer core LPS biosynthesis | (15) |
| *rmlD* | P2095 | JN571483.1 | L5 outer core LPS biosynthesis | (15) |
| *rmlC* | P2095 | JN571483.1 | L5 outer core LPS biosynthesis | (15) |
| *nat_ps* | P2100 | JAMXOU000000000.1 | L6 outer core LPS biosynthesis | (15) |
| *hetA* | P2100 | JAMXOU000000000.1 | L6 outer core LPS biosynthesis | (15) |
| *gatJ* | P2100 | JAMXOU000000000.1 | L6 outer core LPS biosynthesis | (15) |
| *nctB* | P2100 | JAMXOU000000000.1 | L6 outer core LPS biosynthesis | (15) |
| *gatH* | P2100 | JAMXOU000000000.1 | L6 outer core LPS biosynthesis | (15) |
| *gctD* | P2100 | JAMXOU000000000.1 | L6 outer core LPS biosynthesis | (15) |
| *hptE_L6* | P2100 | JAMXOU000000000.1 | L6 outer core LPS biosynthesis | (15) |
| *gatE* | P1581 | JX987237 | L7 outer core LPS biosynthesis | (16) |
| *natA* | P1581 | JX987237 | L7 outer core LPS biosynthesis | (16) |
| *ppgC* | P1581 | JX987237 | L7 outer core LPS biosynthesis | (16) |
| *ppgB* | P1581 | JX987237 | L7 outer core LPS biosynthesis | (16) |
| *ppgA* | P1581 | JX987237 | L7 outer core LPS biosynthesis | (16) |
| *gatC* | P1581 | JX987237 | L7 outer core LPS biosynthesis | (16) |
| *hptE_L8* | P2723 | KM670448 | L8 outer core LPS biosynthesis | (14) |
| *gatM* | P2723 | KM670448 | L8 outer core LPS biosynthesis | (14) |
| *gatK_L8* | P2723 | KM670448 | L8 outer core LPS biosynthesis | (14) |
| *natG* | P2723 | KM670448 | L8 outer core LPS biosynthesis | (14) |
| *natF* | P2723 | KM670448 | L8 outer core LPS biosynthesis | (14) |
| *hetB* | P2723 | KM670448 | L8 outer core LPS biosynthesis | (14) |
| *pfhaB1* | Pm70 | AE004439 | Filamentous haemagglutinin | (10) |
| *pfhaC1* | Pm70 | AE004439 | Filamentous haemagglutinin transporter | (10) |
| *pfhaB2* | Pm70 | AE004439 | Filamentous haemagglutinin 2 | (10) |
| *pfhaC2* | Pm70 | AE004439 | Filamentous haemagglutinin transporter | (10) |
| *fis* | Pm70 | AE004439 | Capsule and virulence factor gene regulator | (10) |
| *hfq* | VP161 | CP048792 | Capsule, LPS transferase and filamentous haemagglutinin gene regulator | (17) |
| *spoT* | VP161 | CP048792 | Caspule gene regulator | (6) |
| *ppx* | VP161 | CP048792 | Caspule gene regulator | (6) |
| *ptsH* | VP161 | CP048792 | Caspule gene regulator | (6) |
| *toxA* | HN06 | CP003313.1 | *Pasteurella multocida*-toxin | (8) |
| *tadG* | HB03 | NZ_CP003328.1 | Flp pili | (18) |
| *tadF* | HB03 | NZ_CP003328.1 | Flp pili | (18) |
| *tadE* | HB03 | NZ_CP003328.1 | Flp pili | (18) |
| *tadD* | HB03 | NZ_CP003328.1 | Flp pili | (18) |
| *tadC* | HB03 | NZ_CP003328.1 | Flp pili | (18) |
| *tadB* | HB03 | NZ_CP003328.1 | Flp pili | (18) |
| *tadA* | HB03 | NZ_CP003328.1 | Flp pili | (18) |
| *tadZ* | HB03 | NZ_CP003328.1 | Flp pili | (18) |
| *rcpB* | HB03 | NZ_CP003328.1 | Flp pili | (18) |
| *rcpA* | HB03 | NZ_CP003328.1 | Flp pili | (18) |
| *rcpC* | HB03 | NZ_CP003328.1 | Flp pili | (18) |
| *tadV* | HB03 | NZ_CP003328.1 | Flp pili | (18) |
| *flp1* | HB03 | NZ_CP003328.1 | Flp pili | (18)  (19) |
| *flp2* | HB03 | NZ_CP003328.1 | Flp pili | (18) |
| *ptfA_1* | Pm70 | AE004439 | Type 4 fimbriae | (10) |
| *ptfA_2* | Pm70 | AE004439 | Type 4 fimbriae | (10) |
| *ompH_1* | Pm70 | AE004439 | Porin | (10) |
| *ompH_2* | Pm70 | AE004439 | Porin | (10) |
| *ompH_3* | Pm70 | AE004439 | Porin | (10) |
| *ompA* | Pm70 | AE004439 | Porin | (10) |
| *oma87* | Pm70 | AE004439 | Porin | (10) |
| *nanH* | Pm70 | AE004439 | Sialic acid scavenging | (10) |
| *nanB* | Pm70 | AE004439 | Sialic acid scavenging | (10) |
| *nanP* | Pm70 | AE004439 | Sialic acid uptake | (10) |
| *nanU* | Pm70 | AE004439 | Sialic acid uptake | (10) |
| *comE1* | Pm70 | AE004439 | Outer membrane protein | (10) |
| *plpB* | Pm70 | AE004439 | Outer membrane protein | (10) |
| *plpE* | Pm70 | AE004439 | Outer membrane protein | (10) |
| *Pmorf0222* | C48-1 | GCA_004286945 | Virulence factor regulator | (20) |
| *Pm0442* | CQ2 | GCF_002891845.2 | Virulence factor regulator | (21) |
| *qseB* | CQ2 | GCF_002891845.2 | Two component regulatory system | (22) |
| *qseC* | CQ2 | GCF_002891845.2 | Two component regulatory system | (22) |
| *lsrA* | Pm70 | AE004439 | Auto inducer-2 quorum sensing system | (10, 23) |
| *lsrB* | Pm70 | AE004439 | Auto inducer-2 quorum sensing system | (10, 23) |
| *lsrC* | Pm70 | AE004439 | Auto inducer-2 quorum sensing system | (10, 23) |
| *lsrD* | Pm70 | AE004439 | Auto inducer-2 quorum sensing system | (10, 23) |
| *lsrE* | Pm70 | AE004439 | Auto inducer-2 quorum sensing system | (10, 23) |
| *lsrF* | Pm70 | AE004439 | Auto inducer-2 quorum sensing system | (10, 23) |
| *lsrG* | Pm70 | AE004439 | Auto inducer-2 quorum sensing system | (10, 23) |
| *lsrK* | Pm70 | AE004439 | Auto inducer-2 quorum sensing system | (10, 23) |
| *lsrR* | Pm70 | AE004439 | Auto inducer-2 quorum sensing system | (10, 23) |
| *luxS* | Pm70 | AE004439 | Auto inducer-2 quorum sensing system | (10, 23) |
| *fur* | Pm70 | AE004439 | Iron aquesition gene regulator | (10) |
| *tbpA* | hh48 | AY007725 | Transferrin binding protein | (24) |
| *pfhR* | Pm70 | AE004439 | Haemoglobin binding protein | (10) |
| *hasR* | Pm70 | AE004439 | Haemoglobin binding protein | (10) |
| *hemR* | Pm70 | AE004439 | Haemoglobin binding protein | (10) |
| *hgbA* | Pm70 | AE004439 | Haemoglobin binding protein | (10) |
| *hgbB* | Pm70 | AE004439 | Haemoglobin binding protein | (10) |
| *hpbA* | Pm70 | AE004439 | Haemin binding protein | (10) |
| *Pm0336* | Pm70 | AE004439 | Haemoglobin binding protein | (10) |
| *Pm0741* | Pm70 | AE004439 | Haemoglobin binding protein | (10) |
| *Pm1081* | Pm70 | AE004439 | Haemoglobin binding protein | (10) |
| *Pm1282* | Pm70 | AE004439 | Haemoglobin binding protein | (10) |
| *Pm1428* | Pm70 | AE004439 | Haemoglobin binding protein | (10) |
| *tonB* | Pm70 | AE004439 | TonB system | (10) |
| *exbB* | Pm70 | AE004439 | TonB system | (10) |
| *exbD* | Pm70 | AE004439 | TonB system | (10) |
| *afuA_1* | Pm70 | AE004439 | Iron uptake ABC-transporter | (10) |
| *afuA_2* | Pm70 | AE004439 | Iron uptake ABC-transporter | (10) |
| *afuA_3* | Pm70 | AE004439 | Iron uptake ABC-transporter | (10) |
| *afuB* | Pm70 | AE004439 | Iron uptake ABC-transporter | (10) |
| *afuC* | Pm70 | AE004439 | Iron uptake ABC-transporter | (10) |
| *fbpA* | Pm70 | AE004439 | Iron uptake ABC-transporter | (10) |
| *fbpB* | Pm70 | AE004439 | Iron uptake ABC-transporter | (10) |
| *fbpC* | Pm70 | AE004439 | Iron uptake ABC-transporter | (10) |
| *fecB* | Pm70 | AE004439 | Iron uptake ABC-transporter | (10) |
| *fecC* | Pm70 | AE004439 | Iron uptake ABC-transporter | (10) |
| *fecD* | Pm70 | AE004439 | Iron uptake ABC-transporter | (10) |
| *fecE* | Pm70 | AE004439 | Iron uptake ABC-transporter | (10) |
| *yfeA* | Pm70 | AE004439 | Iron uptake ABC-transporter | (10) |
| *yfeB* | Pm70 | AE004439 | Iron uptake ABC-transporter | (10) |
| *yfeC* | Pm70 | AE004439 | Iron uptake ABC-transporter | (10) |
| *yfeD* | Pm70 | AE004439 | Iron uptake ABC-transporter | (10) |
| *mobA_pB1000* | pB1000 | DQ840517 | Mobilisation protein | (25) |
| *mobB_pB1000* | pB1000 | DQ840517 | Mobilisation protein | (25) |
| *mobC_pB1000* | pB1000 | DQ840517 | Mobilisation protein | (25) |
| *mobA_pCCK1900* | pCCK1900 | FM179941 | Mobilisation protein | (26) |
| *mobB_pCCK1900* | pCCK1900 | FM179941 | Mobilisation protein | (26) |
| *mobC_pCCK1900* | pCCK1900 | FM179941 | Mobilisation protein | (26) |
| *repA* | pCCK1900 | FM179941 | Plasmid replication protein | (26) |
| *repB* | pCCK1900 | FM179941 | Plasmid replication protein | (26) |
| *repC* | pCCK1900 | FM179941 | Plasmid replication protein | (26) |
| *blaROB-1* | pB1000 | DQ840517 | β-lactam resistance | (25) |
| *tetH* | p9956 | AY362554 | Tetracycline resistance | (25, 27) |
| *tetR* | p9956 | AY362554 | Tetracycline repressor protein | (25, 27) |
| *tetO* | pB1006 | FJ234438 | Tetracycline resistance | (25) |
| *tetB* | pB1001 | EU252517 | Tetracycline resistance | (25) |
| *rep_pB1001* | pB1001 | EU252517 | Plasmid replication protein | (25) |
| *tetG* | pJR1 | AY232670.1 | Tetracycline resistance | (28) |
| *catB2* | pJR1 | AY232670.1 | Chloramphenicol resistance | (28) |
| *rep_pJR1* | pJR1 | AY232670.1 | Plasmid replication protein | (28) |
| *aadA1* | pJR2 | AY232671 | Streptomycin/Spectinomycin resistance | (28) |
| *blaP1* | pJR2 | AY232671 | β-lactam resistance | (28) |
| *rep_pJR2* | pJR2 | AY232671 | Plasmid replication protein | (28) |
| *floR* | pCCK1900 | FM179941 | Florfenicol resistance | (26) |
| *sul2* | pCCK1900 | FM179941 | Sulfonamide resistance | (26) |
| *strA* | pCCK1900 | FM179941 | Streptomycin/Spectinomycin resistance | (26) |
| *strB* | pCCK1900 | FM179941 | Streptomycin/Spectinomycin resistance | (26) |
| *aadA14* | pCCK647 | AJ884726 | Streptomycin/Spectinomycin resistance | (29) |
| *rep_pCCK647* | pCCK647 | AJ884726 | Plasmid replication protein | (29) |
| *dfrA14* | pIMT41689_PM | MH910619 | Trimethoprim/Sulfamethoxazole resistance | (30) |
| *cfr* | pFJ6683 | MT338505 | Multiclass antibiotic resistance | (31, 32) |
| *blaVEB-1* | pFJ6683 | MT338505 | β-lactam resistance | (31, 32) |
| *aadB* | pFJ6683 | MT338505 | Aminoglycoside resistance | (31, 32) |
| *arr2* | pFJ6683 | MT338505 | Rifampin resistance | (31, 32) |
| *cmlA* | pFJ6683 | MT338505 | Chloramphenicol resistance | (31, 32) |
| *aphA3* | pCCK411 | FR798946 | Kanamycin resistance | (33) |
| *dfrA20* | pCCK154 | AJ605332 | Trimethoprim resistance | (34) |
| *int1* | 36950 | CP003022 | ICE*Pmu1* integrase 1 | (35, 36) |
| *int2* | 36950 | CP003022 | ICE*Pmu1* intergrase2 | (35, 36) |
| *rel* | 36950 | CP003022 | ICE*Pmu1* relaxase | (35, 36) |
| *parA* | 36950 | CP003022 | ICE*Pmu1* mantenance | (35-38) |
| *dnaB1* | 36950 | CP003022 | ICE*Pmu1* mantenance, DNA helicase | (35-38) |
| *parB* | 36950 | CP003022 | ICE*Pmu1* mantenance | (35-38) |
| *topB1* | 36950 | CP003022 | ICE*Pmu1* mantenance, topoisomerase | (35-38) |
| *traC* | 36950 | CP003022 | ICE*Pmu1* conjugation | (35-38) |
| *traD* | 36950 | CP003022 | ICE*Pmu1* conjugation | (35-38) |
| *ISApl1* | 36950 | CP003022 | Transposase | (35, 36) |
| *ISCR21* | 36950 | CP003022 | Transposase | (35, 36) |
| *ISCR2* | 36950 | CP003022 | Transposase | (35, 36) |
| *IS26* | 36950 | CP003022 | Transposase | (35, 36) |
| *tnpA* | 36950 | CP003022 | Transposase | (35, 36) |
| *mphE* | 36950 | FR751518.1 | Macrolide/Triamilide resistance | (39) |
| *mrsE* | 36950 | FR751518.1 | Macrolide/Triamilide resistance | (39) |
| *erm42* | 36950 | FR734406.1 | Macrolide/Lincosamide resistance | (39) |
| *aphA1* | 36950 | CP003022 | Kanamycin resistance | (35, 36) |
| *aadA25* | 36950 | CP003022 | Streptomycin/Spectinomycin resistance | (35, 36) |
| *blaOXA2* | 36950 | CP003022 | β-lactam resistance | (35, 36) |
| *tfc6* | HN07 | CP007040 | ICE*pmcn07* conjugation | (40, 41) |
| *tfc14* | HN07 | CP007040 | ICE*pmcn07* conjugation | (40, 41) |
| *tfc16* | HN07 | CP007040 | ICE*pmcn07* conjugation | (40, 41) |

^1^Genes included as a genome location reference


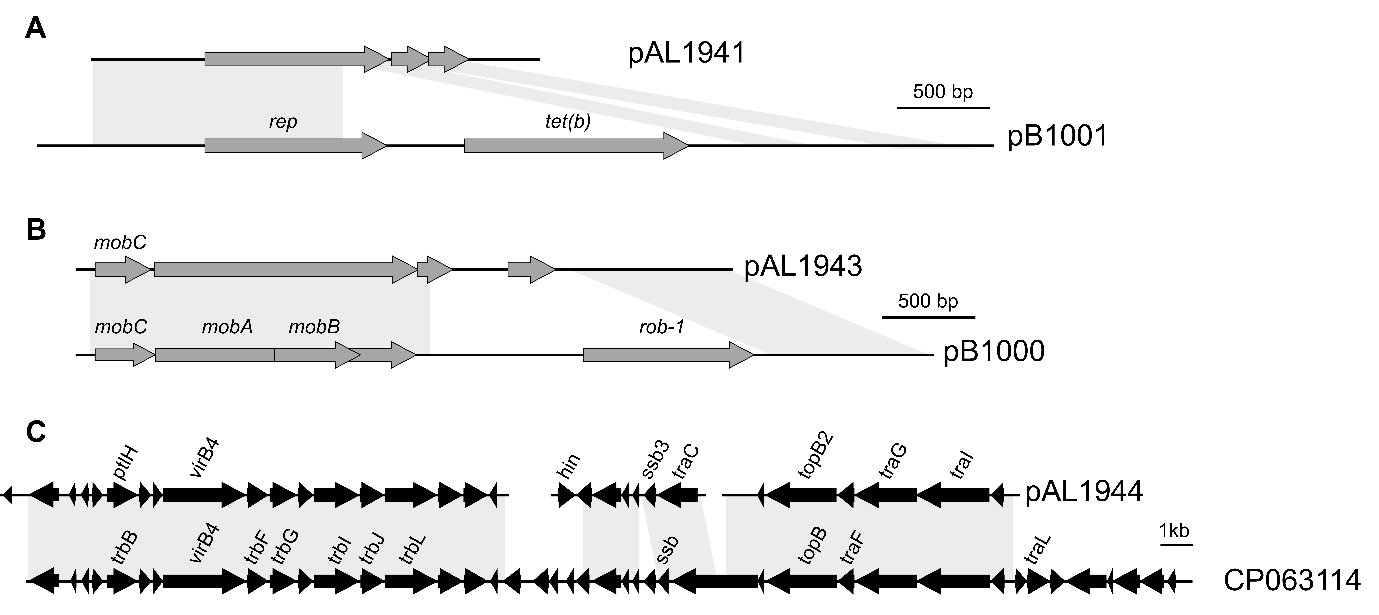


**FIG S1**. Plasmids pAL1941, pAL1943 and pAL1944, identified in *P. multocida* strains sequenced in this study. Gene names are given where possible, unnamed genes encode proteins with no known function. Grey shaded areas indicate regions of homology identified by Mauve for pAL1941 and pB1001 **(A)**, pAL1943 and pB1000 **(B)**, and pAL1944 contigs and a plasmid from *Haemophilus influenzae* strain M1C147_1 **(C)**.

**
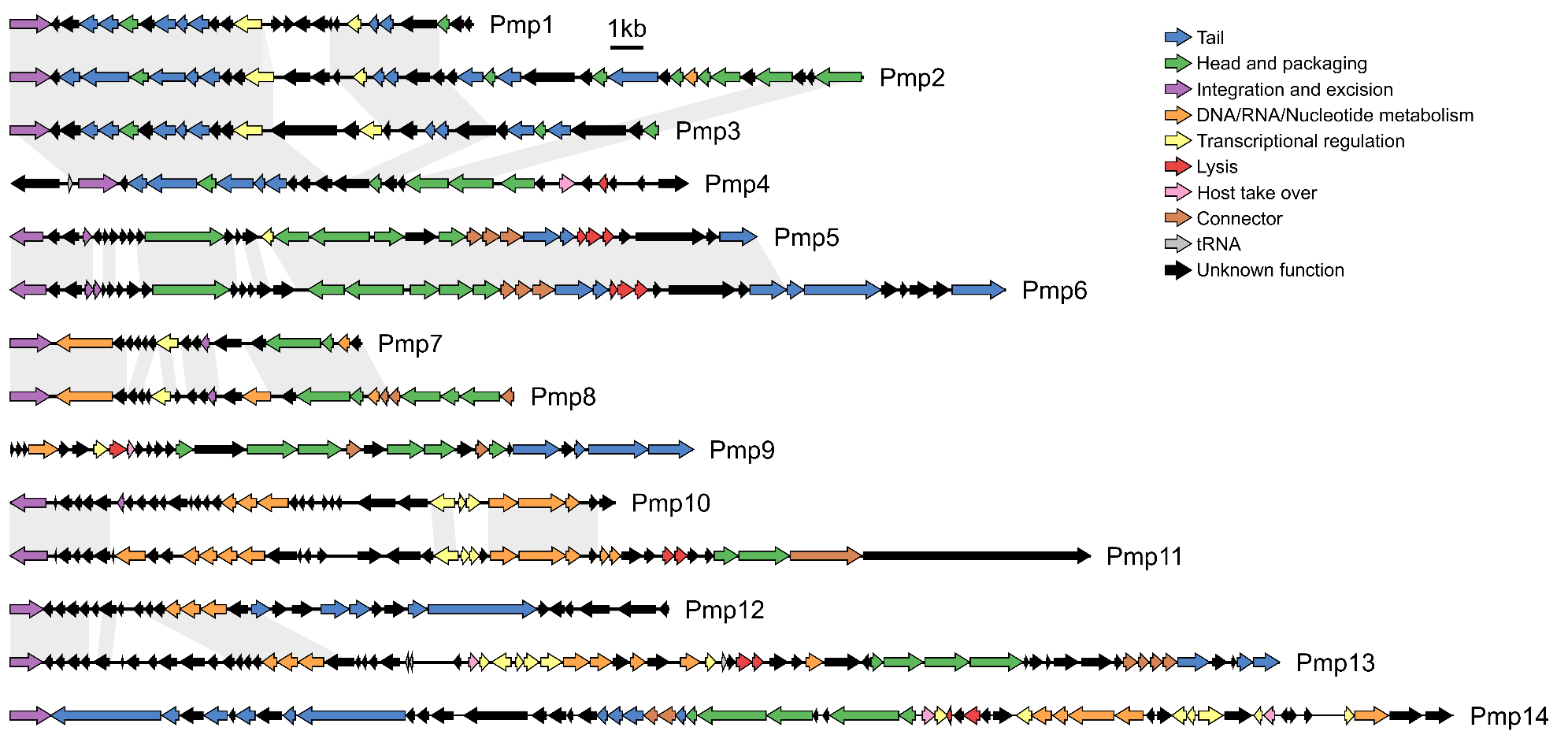
**

**FIG S2.** Genetic maps of the 14 prophages identified in the *P. multocida* strains sequenced in this study. Prophages were identified using PhiSpy, and the general function of open reading frames was identified using Pharokka, with general function of open reading frame products represented by colour. Grey shaded areas represent regions of homology identified by Mauve between different prophages identified in this study.


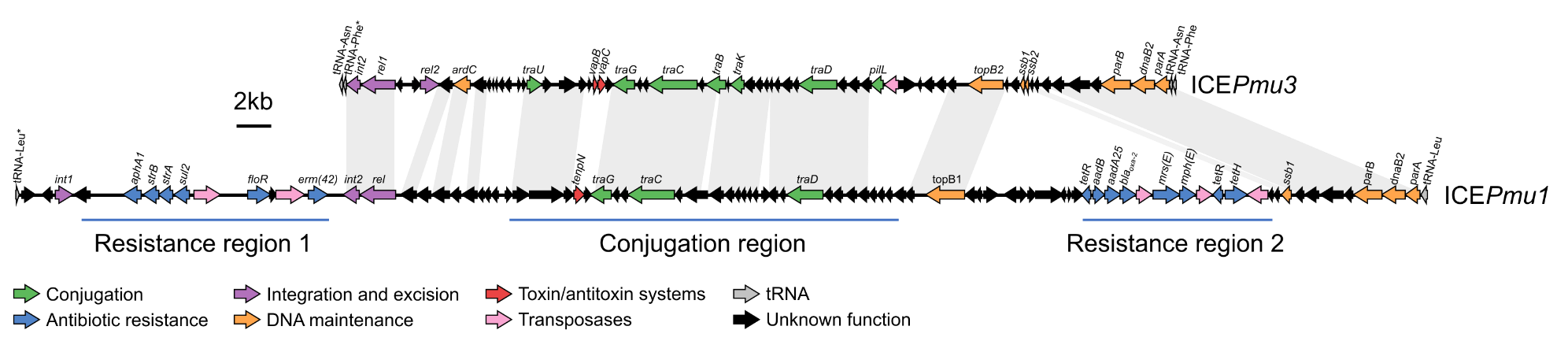


**FIG S3.** Genetic map of ICE*Pmu3* identified in *P. multocida* strains Past26 and Past31. ICE*Pmu1*, identified in *P. multocida* strain 36950 is included as a comparison. General function of open reading frame products are represented by colour, with the legend below. Grey shaded areas represent regions of homology identified by Mauve between ICE*Pmu3* and ICE*Pmu1*.

**
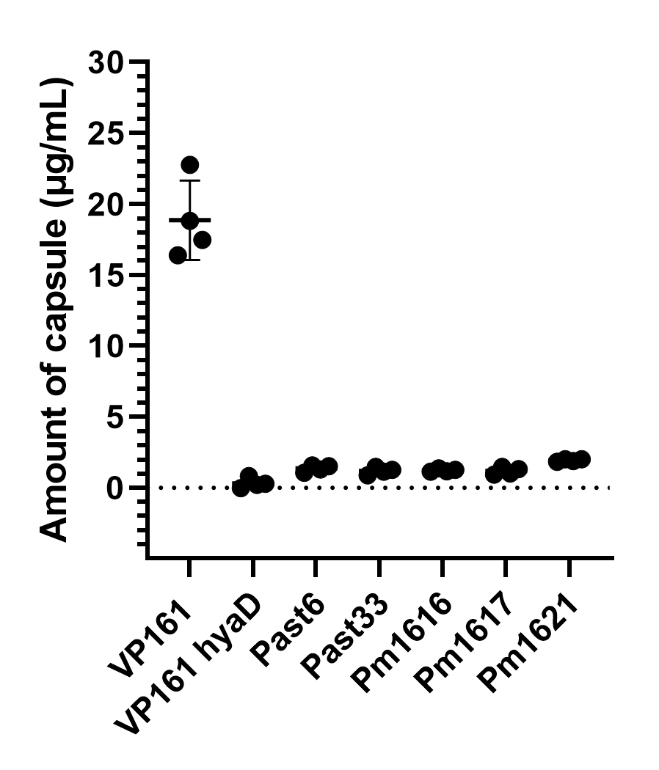
**

**FIG S4.** Amount of capsule extracted from different *P. multocida* strains measured using a capsule quantification assay. Measurements were performed using the *P. multocida* strains isolated from humans, cats, and dogs that did not have a capsule locus, *P. multocida* strain VP161 that produces a hyaluronic acid capsule, and a VP161 *hyaD* TargeTron mutant strain that is known to not produce capsule. Capsule was extracted from strains in biological quadruplicate from mid-exponential-phase growth cultures. Error bars represent mean ± standard deviation (SD). Amount of capsule was determined using a standard curve generated using hyaluronic acid of known concentrations.


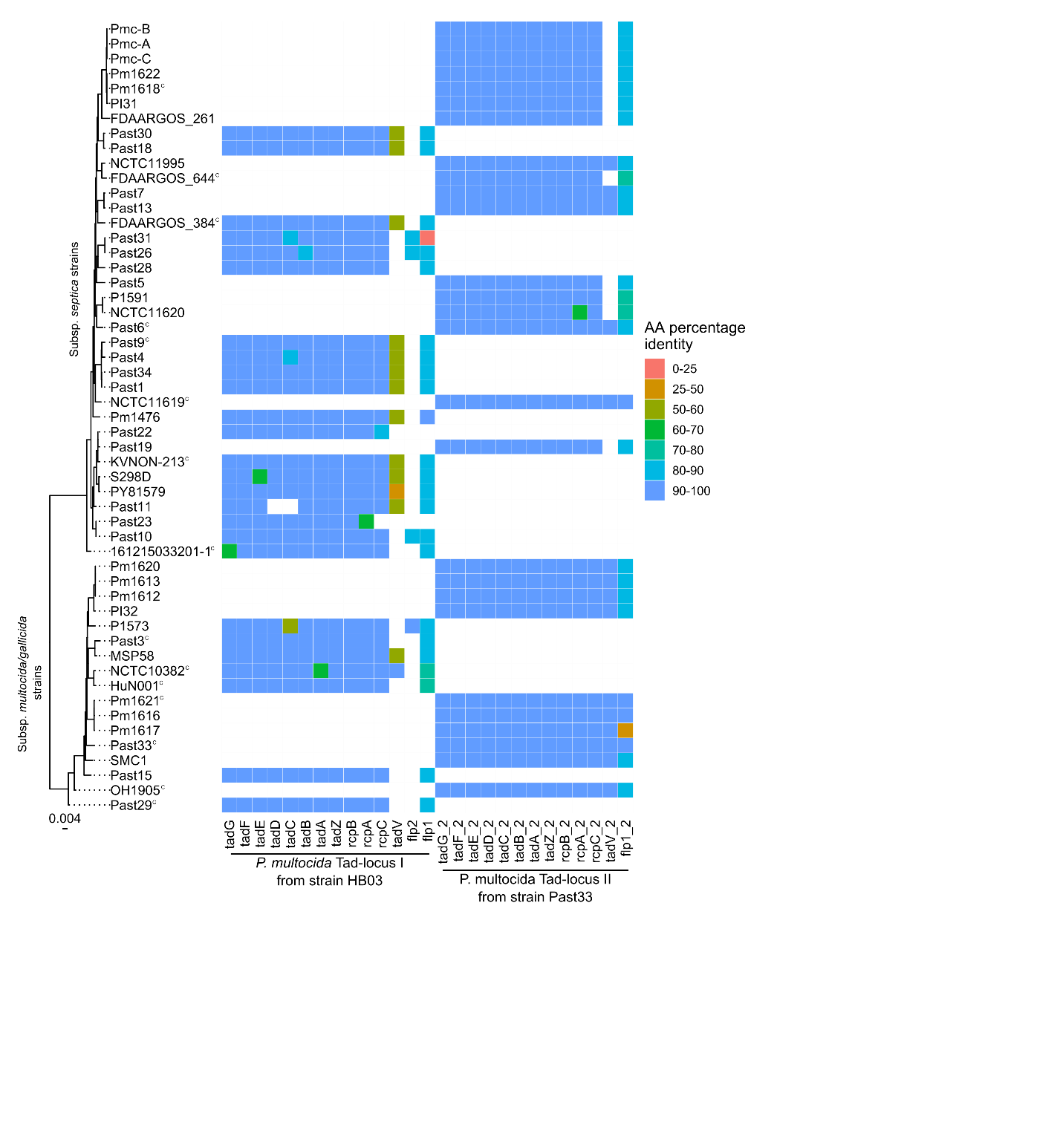


**FIG S5.** Heatmap showing the presence of genes in the two Tad-loci in *P. multocida* strains isolated from humans, cats, and dogs. Genome sequences of the *P. multocida* strains were searched against the *P. multocida* virulence factor and antibiotic resistance using Assembly2Feature. Coloured squares represent relative amino acid identity between proteins encoded by query and reference sequences. For reference, a maximum-likelihood core-genome phylogeny of *P. multocida* isolates recovered from humans, cats, and dogs is shown. The core-genome alignment was generated using Roary, and the maximum-likelihood tree was generated using IQ-TREE modelled with empirical base frequencies and a FreeRate model distribution allowing for a proportion of invariable sites (GTR+F+I+G4), with 1,000 bootstrap replicates. Scale bar represents number of nucleotide substitutions per site. Both closed and incomplete genomes were included in the analysis, with closed genomes indicated by a superscript C.

References

1. Jain C, Rodriguez RL, Phillippy AM, Konstantinidis KT, Aluru S. 2018. High throughput ANI analysis of 90K prokaryotic genomes reveals clear species boundaries. Nat Commun 9:5114.

2. Pearce ME, Langridge GC, Lauer AC, Grant K, Maiden MCJ, Chattaway MA. 2021. An evaluation of the species and subspecies of the genus *Salmonella* with whole genome sequence data: Proposal of type strains and epithets for novel *S. enterica* subspecies VII, VIII, IX, X and XI. Genomics 113:3152-3162.

3. Minias A, Żukowska L, Lach J, Jagielski T, Strapagiel D, Kim SY, Koh WJ, Adam H, Bittner R, Truden S, Žolnir-Dovč M, Dziadek J. 2020. Subspecies-specific sequence detection for differentiation of *Mycobacterium abscessus* complex. Sci Rep 10:16415.

4. Ujvári B, Gantelet H, Magyar T. 2022. Development of a multiplex PCR assay for the detection of key genes associated with *Pasteurella multocida* subspecies. J Vet Diagn Invest 34:319-322.

5. Wilkie IW, Grimes SE, O'Boyle D, Frost AJ. 2000. The virulence and protective efficacy for chickens of *Pasteurella multocida* administered by different routes. Vet Microbiol 72:57-68.

6. Smallman TR, Williams GC, Harper M, Boyce JD. 2022. Genome-wide investigation of *Pasteurella multocida* identifies the stringent response as a negative regulator of hyaluronic acid capsule production. Microbiol Spectr 10:e0019522.

7. Townsend KM, Boyce JD, Chung JY, Frost AJ, Adler B. 2001. Genetic organization of *Pasteurella multocida* cap Loci and development of a multiplex capsular PCR typing system. J Clin Microbiol 39:924-9.

8. Liu W, Yang M, Xu Z, Zheng H, Liang W, Zhou R, Wu B, Chen H. 2012. Complete genome sequence of *Pasteurella multocida* HN06, a toxigenic strain of serogroup D. J Bacteriol 194:3292-3.

9. Boyce JD, Adler B. 2000. The capsule is a virulence determinant in the pathogenesis of *Pasteurella multocida* M1404 (B:2). Infect Immun 68:3463-8.

10. May BJ, Zhang Q, Li LL, Paustian ML, Whittam TS, Kapur V. 2001. Complete genomic sequence of *Pasteurella multocida*, Pm70. Proc Natl Acad Sci USA 98:3460-5.

11. Harper M, St Michael F, John M, Steen J, van Dorsten L, Parnas H, Vinogradov E, Adler B, Cox AD, Boyce JD. 2014. Structural analysis of lipopolysaccharide produced by Heddleston serovars 10, 11, 12 and 15 and the identification of a new *Pasteurella multocida* lipopolysaccharide outer core biosynthesis locus, L6. Glycobiology 24:649-59.

12. St Michael F, Harper M, Parnas H, John M, Stupak J, Vinogradov E, Adler B, Boyce JD, Cox AD. 2009. Structural and genetic basis for the serological differentiation of *Pasteurella multocida* Heddleston serotypes 2 and 5. J Bacteriol 191:6950-9.

13. Harper M, St Michael F, John M, Vinogradov E, Steen JA, van Dorsten L, Steen JA, Turni C, Blackall PJ, Adler B, Cox AD, Boyce JD. 2013. *Pasteurella multocida* Heddleston serovar 3 and 4 strains share a common lipopolysaccharide biosynthesis locus but display both inter- and intrastrain lipopolysaccharide heterogeneity. J Bacteriol 195:4854-64.

14. Harper M, St Michael F, Steen JA, John M, Wright A, van Dorsten L, Vinogradov E, Adler B, Cox AD, Boyce JD. 2015. Characterization of the lipopolysaccharide produced by *Pasteurella multocida* serovars 6, 7 and 16: identification of lipopolysaccharide genotypes L4 and L8. Glycobiology 25:294-302.

15. Harper M, John M, Turni C, Edmunds M, St Michael F, Adler B, Blackall PJ, Cox AD, Boyce JD. 2015. Development of a rapid multiplex PCR assay to genotype *Pasteurella multocida* strains by use of the lipopolysaccharide outer core biosynthesis locus. J Clin Microbiol 53:477-85.

16. Harper M, St Michael F, Vinogradov E, John M, Steen JA, van Dorsten L, Boyce JD, Adler B, Cox AD. 2013. Structure and biosynthetic locus of the lipopolysaccharide outer core produced by *Pasteurella multocida* serovars 8 and 13 and the identification of a novel phospho-glycero moiety. Glycobiology 23:286-94.

17. Mégroz M, Kleifeld O, Wright A, Powell D, Harrison P, Adler B, Harper M, Boyce JD. 2016. The RNA-binding chaperone Hfq is an important global regulator of gene expression in *Pasteurella multocida* and plays a crucial role in production of a number of virulence factors, including hyaluronic acid capsule. Infect Immun 84:1361-1370.

18. Peng Z, Wang X, Zhou R, Chen H, Wilson BA, Wu B. 2019. *Pasteurella multocida*: genotypes and genomics. Microbiol Mol Biol Rev 83.

19. Harper M, Boyce JD, Wilkie IW, Adler B. 2003. Signature-tagged mutagenesis of *Pasteurella multocida* identifies mutants displaying differential virulence characteristics in mice and chickens. Infect Immun 71:5440-6.

20. Xu T, Zheng Y, Liu B, Kou M, Jiang Q, Liu J, Kang H, Yang M, Guo D, Qu L. 2023. Pmorf0222, a virulence factor in *Pasteurella multocida*, activates nuclear factor kappa B and mitogen-activated protein kinase via toll-like receptor 1/2. Infect Immun 91:e0019322.

21. He F, Qin X, Xu N, Li P, Wu X, Duan L, Du Y, Fang R, Hardwidge PR, Li N, Peng Y. 2020. *Pasteurella multocida* Pm0442 affects virulence gene expression and targets TLR2 to induce inflammatory responses. Front Microbiol 11:1972.

22. Yang Y, Hu P, Gao L, Yuan X, Hardwidge PR, Li T, Li P, He F, Peng Y, Li N. 2021. Deleting qseC downregulates virulence and promotes cross-protection in *Pasteurella multocida*. Vet Res 52:140.

23. Malott RJ, Lo RY. 2002. Studies on the production of quorum-sensing signal molecules in *Mannheimia haemolytica* A1 and other Pasteurellaceae species. FEMS Microbiol Lett 206:25-30.

24. Ogunnariwo JA, Schryvers AB. 2001. Characterization of a novel transferrin receptor in bovine strains of *Pasteurella multocida*. J Bacteriol 183:890-6.

25. San Millan A, Escudero JA, Gutierrez B, Hidalgo L, Garcia N, Llagostera M, Dominguez L, Gonzalez-Zorn B. 2009. Multiresistance in *Pasteurella multocida* is mediated by coexistence of small plasmids. Antimicrob Agents Chemother 53:3399-404.

26. Kehrenberg C, Wallmann J, Schwarz S. 2008. Molecular analysis of florfenicol-resistant *Pasteurella multocida* isolates in Germany. J Antimicrob Chemother 62:951-5.

27. Blanco M, Kadlec K, Gutiérrez Martín CB, de la Fuente AJ, Schwarz S, Navas J. 2007. Nucleotide sequence and transfer properties of two novel types of *Actinobacillus pleuropneumoniae* plasmids carrying the tetracycline resistance gene *tet(H)*. J Antimicrob Chemother 60:864-7.

28. Wu JR, Shieh HK, Shien JH, Gong SR, Chang PC. 2003. Molecular characterization of plasmids with antimicrobial resistant genes in avian isolates of *Pasteurella multocida*. Avian Dis 47:1384-92.

29. Kehrenberg C, Catry B, Haesebrouck F, de Kruif A, Schwarz S. 2005. Novel spectinomycin/streptomycin resistance gene, *aadA14*, from *Pasteurella multocida*. Antimicrob Agents Chemother 49:3046-9.

30. Niemann L, Feudi C, Eichhorn I, Hanke D, Müller P, Brauns J, Nathaus R, Schäkel F, Höltig D, Wendt M, Kadlec K, Schwarz S. 2019. Plasmid-located *dfrA14* gene in *Pasteurella multocida* isolates from three different pig-producing farms in Germany. Vet Microbiol 230:235-240.

31. Chen H, Deng H, Cheng L, Jiang N, Fu G, Shi S, Wan C, Fu Q, Liu R, Huang X, Huang Y. 2022. Complete nucleotide sequence of cfr-harbouring multidrug-resistant plasmid, pFJ6683, from avian *Pasteurella multocida*. J Antimicrob Chemother 77:3517-3519.

32. Chen H, Deng H, Cheng L, Liu R, Fu G, Shi S, Wan C, Fu Q, Huang Y, Huang X. 2020. First report of the multiresistance gene cfr in *Pasteurella multocida* strains of avian origin from China. J Glob Antimicrob Resist 23:251-255.

33. Yamashita A, Sekizuka T, Kuroda M. 2014. Characterization of antimicrobial resistance dissemination across plasmid communities classified by network analysis. Pathogens 3:356-76.

34. Kehrenberg C, Schwarz S. 2005. *dfrA20*, A novel trimethoprim resistance gene from *Pasteurella multocida*. Antimicrob Agents Chemother 49:414-7.

35. Michael GB, Kadlec K, Sweeney MT, Brzuszkiewicz E, Liesegang H, Daniel R, Murray RW, Watts JL, Schwarz S. 2012. ICE*Pmu1*, an integrative conjugative element (ICE) of *Pasteurella multocida*: analysis of the regions that comprise 12 antimicrobial resistance genes. J Antimicrob Chemother 67:84-90.

36. Michael GB, Kadlec K, Sweeney MT, Brzuszkiewicz E, Liesegang H, Daniel R, Murray RW, Watts JL, Schwarz S. 2012. ICE*Pmu1*, an integrative conjugative element (ICE) of *Pasteurella multocida*: structure and transfer. J Antimicrob Chemother 67:91-100.

37. Cameron A, Zaheer R, McAllister TA. 2019. Emerging variants of the integrative and conjugant element ICE*Mh1* in livestock pathogens: structural insights, potential host range, and implications for bacterial fitness and antimicrobial therapy. Front Microbiol 10:2608.

38. Beker M, Rose S, Lykkebo CA, Douthwaite S. 2018. Integrative and conjugative Elements (ICEs) in Pasteurellaceae species and their detection by multiplex PCR. Front Microbiol 9:1329.

39. Kadlec K, Brenner Michael G, Sweeney MT, Brzuszkiewicz E, Liesegang H, Daniel R, Watts JL, Schwarz S. 2011. Molecular basis of macrolide, triamilide, and lincosamide resistance in *Pasteurella multocida* from bovine respiratory disease. Antimicrob Agents Chemother 55:2475-7.

40. Peng Z, Liang W, Wang Y, Liu W, Zhang H, Yu T, Zhang A, Chen H, Wu B. 2017. Experimental pathogenicity and complete genome characterization of a pig origin *Pasteurella multocida* serogroup F isolate HN07. Vet Microbiol 198:23-33.

41. Juhas M, Crook DW, Dimopoulou ID, Lunter G, Harding RM, Ferguson DJ, Hood DW. 2007. Novel type IV secretion system involved in propagation of genomic islands. J Bacteriol 189:761-71.
